# Approach and Withdrawal Tendencies during Written Word Processing: Effects of Task, Emotional Valence, and Emotional Arousal

**DOI:** 10.3389/fpsyg.2015.01935

**Published:** 2016-01-06

**Authors:** Francesca M. M. Citron, David Abugaber, Cornelia Herbert

**Affiliations:** ^1^Department of Psychology, Lancaster UniversityLancaster, UK; ^2^Cluster of Excellence “Languages of Emotion”, Free University of BerlinBerlin, Germany; ^3^Psychology Department, Humanities Council, Princeton UniversityPrinceton, NJ, USA; ^4^Department of Theoretical and Applied Linguistics, University of CambridgeCambridge, UK; ^5^Applied Emotion and Motivation Research, Institute of Psychology and Education, Ulm UniversityUlm, Germany; ^6^Department of Psychiatry, University of TübingenTübingen, Germany; ^7^Department of Biomedical Magnetic Resonance Imaging, University of TübingenTübingen, Germany; ^8^Department of Psychology, University of WürzburgWürzburg, Germany

**Keywords:** approach, withdrawal, valence, arousal, emotion, words, polarity effects

## Abstract

The affective dimensions of emotional valence and emotional arousal affect processing of verbal and pictorial stimuli. Traditional emotional theories assume a linear relationship between these dimensions, with valence determining the direction of a behavior (approach vs. withdrawal) and arousal its intensity or strength. In contrast, according to the valence-arousal conflict theory, both dimensions are interactively related: positive valence and low arousal (PL) are associated with an implicit tendency to approach a stimulus, whereas negative valence and high arousal (NH) are associated with withdrawal. Hence, positive, high-arousal (PH) and negative, low-arousal (NL) stimuli elicit conflicting action tendencies. By extending previous research that used several tasks and methods, the present study investigated whether and how emotional valence and arousal affect subjective approach vs. withdrawal tendencies toward emotional words during two novel tasks. In Study 1, participants had to decide whether they would approach or withdraw from concepts expressed by written words. In Studies 2 and 3 participants had to respond to each word by pressing one of two keys labeled with an arrow pointing upward or downward. Across experiments, positive and negative words, high or low in arousal, were presented. In Study 1 (explicit task), in line with the valence-arousal conflict theory, PH and NL words were responded to more slowly than PL and NH words. In addition, participants decided to approach positive words more often than negative words. In Studies 2 and 3, participants responded faster to positive than negative words, irrespective of their level of arousal. Furthermore, positive words were significantly more often associated with “up” responses than negative words, thus supporting the existence of implicit associations between stimulus valence and response coding (positive is up and negative is down). Hence, in contexts in which participants' spontaneous responses are based on implicit associations between stimulus valence and response, there is no influence of arousal. In line with the valence-arousal conflict theory, arousal seems to affect participants' approach-withdrawal tendencies only when such tendencies are made explicit by the task, and a minimal degree of processing depth is required.

## Introduction

According to dimensional models of emotion, valence describes the extent to which a stimulus is positive or negative whereas emotional arousal refers to its degree of physiological activation, i.e., how calming or exciting/agitating a stimulus is (Russell, 1980, 2003; Reisenzein, 1994; Lang et al., 1997). This two-dimensional approach to emotions originates from work by Osgood et al. (1957) in which large word samples were rated on several stimulus dimensions that could methodologically be reduced to three underlying common factors: emotional evaluation (i.e., valence), potency (i.e., arousal), and activity (i.e., dominance). The first two dimensions could account for most of the variance in ratings. When stimuli are mapped in affective space according to their subjective ratings, emotional valence and arousal ratings typically show a quadratic relationship, whereby highly positive or negative stimuli are also rated higher in their level of arousal; furthermore, negative stimuli tend to be rated higher in arousal than positive stimuli (Bradley and Lang, 1999; Lang et al., 1999; Võ et al., 2009; Montefinese et al., 2013; Citron et al., 2014b). Despite this relationship, the two dimensions of valence and arousal are considered distinct affective dimensions; in fact, they are associated with different physiological and affective behavioral responses (Lang et al., 1990, 1993), activate partially-dissociable brain networks (Small et al., 2003; Lewis et al., 2007; Wilson-Mendenhall et al., 2013) and are correlated with different lexico-semantic properties such as familiarity, imageability, and concreteness (Kousta et al., 2011; Montefinese et al., 2013; Citron et al., 2014b; Schmidtke et al., 2014). In line with this dimensional view, empirical research has shown that a wide range of emotional stimuli including pictures, faces, words (denoting emotions, personality traits, or other concepts), and even short scenarios describing specific emotions, can be successfully mapped onto this two-dimensional affective space and distinguished by their position within that space (Abelson and Sermant, 1962; Russell, 1980; Cacioppo and Berntson, 1994; Barrett and Russell, 1999; Lang et al., 1999; Wilson-Mendenhall et al., 2013).

Crucially, it has been proposed that the position within the affective space determined by the two major dimensions of valence and arousal reflects the activation of two motivational systems of approach and withdrawal: positive valence elicits the activation of the approach system and negative valence the activation of the withdrawal system. In contrast to valence, the arousal dimension indicates the intensity of physiological activation in either of the two systems (for an overview, see Lang et al., 1997).

In recent years, several studies aimed to find behavioral evidence for this assumption by investigating the processing of highly arousing positive and negative stimuli compared to neutral stimuli, either during viewing of emotional pictures or reading of emotional words. These included reaction time (RT) studies (e.g., Algom et al., 2004; Larsen et al., 2006; Kousta et al., 2009; Nasrallah et al., 2009) as well as studies on motivational priming of the startle reflex during picture viewing or word processing (e.g., Herbert et al., 2006, 2011, 2014; Herbert and Kissler, 2010; for an overview, see Lang et al., 1997; Bradley et al., 2006). The startle reflex and its affective modulation is one of the most prominent and basic bio-psychological measures of approach and withdrawal. In sum, research on affective startle reflex modulation suggested that the higher a stimulus is rated on the emotional arousal dimension, the stronger the physiological motivation to approach or withdraw from it (Lang et al., 1997). This suggests that stimulus valence and arousal do have additive effects on affective processing, such that responses to positive and negative stimuli should be more pronounced if their level of arousal is high.

However, Robinson et al. (2004) suggested a different view: based on their own empirical observations, they proposed the valence-arousal conflict theory and suggest that the two affective dimensions of a stimulus (i.e., its valence and its arousal) automatically elicit a specific response tendency independently from the other. In particular, they proposed that, independently from their valence, stimuli rated high in emotional arousal will be appraised as negative/unpleasant, whereas stimuli rated as low in stimulus arousal will be more likely appraised as positive/appetitive, thereby producing a conflict in responding if stimulus valence and arousal do not match, i.e., positive valence and high emotional arousal (PH) or negative valence and low emotional arousal (NL). So far, there exist a number of behavioral studies to support this assumption. For instance, Robinson and colleagues themselves demonstrated, in several experiments employing emotional pictures or words, that RTs to conflicting stimuli (PH and NL) are slower in comparison to stimuli producing no conflict, i.e., positive valence, low arousal (PL) and negative valence, high arousal (NH). In these studies, participants had to appraise the valence of the stimulus in a valence judgment task (positive vs. negative) (see also Eder and Rothermund, 2010). Similar effects have been reported in an arousal judgment task (high vs. low), using pictures as well as words (Purkis et al., 2009). These tasks explicitly direct the attention to the emotional content of the stimuli. However, similar effects have also been reported during more implicit tasks such as color judgment (Feng et al., 2012), the affective Simon task (Eder and Rothermund, 2010), or the lexical decision task (LDT; Larsen et al., 2008; Hofmann et al., 2009; Eder and Rothermund, 2010; Feng et al., 2012; Citron et al., 2014c).

Together, these findings suggest that stimulus valence and stimulus arousal both influence participants' evaluation of emotional stimuli across a variety of tasks; this does not occur linearly or in an additive way as predicted by traditional emotion models, but interactively, as suggested by Robinson et al.'s conflict theory. This interactive view is also suggested by neuroscientific studies that showed significant processing differences between high- and low-arousal positive and negative stimuli already during early stages of cortical processing, associated with sensory stimulus processing and attention orientation (Hofmann et al., 2009; Feng et al., 2012; Citron et al., 2013; Recio et al., 2014). Furthermore, there is some evidence that brain regions associated with the integration of physiological and cognitive responses such as the insula are modulated differently by high- and low-arousal positive and negative stimuli (Citron et al., 2014a).

Nevertheless, it is still an open issue whether differential processing effects reported for PH and NL vs. PL and NH stimuli in previous studies are actually related to approach and withdrawal tendencies. In fact, none of the aforementioned studies that investigated the preparation of approach and withdrawal tendencies by using biological measures and paradigms such as the affective startle modulation found evidence in favor of this thesis. Furthermore, some empirical evidence suggests a higher cognitive accessibility of the valence than the arousal dimension (Nicolle and Goel, 2012). In addition, studies that used more response-focused tasks such as the stop-signal task in combination with verbal material suggest serial effects of perception, attention and action (e.g., Herbert and Sütterlin, 2012), which contrasts with the idea that approach and withdrawal tendencies are primed effortlessly before a minimal degree of linguistic processing has taken place. However, according to Robinson et al. (2004), PH and NL stimuli are expected to elicit conflicting response tendencies that slow down RTs irrespective of whether the task requires stimulus evaluation or a motor response (e.g., a key press associated with moving one's finger or hand forward or backward; see Robinson et al., 2004, Study 4). This assumption should hold for a broad range of stimuli including abstract and symbolic stimuli such as words, which have been shown to evoke similar affective responses as pictures (Citron, 2012; Tempel et al., 2013).

Building upon and extending these previous findings, the current study aimed to address two main research questions: (1) Do high- and low-arousal, positive and negative stimuli elicit differential responses when these are explicitly associated with approach and withdrawal tendencies? (2) Will these effects arise even in an implicit task that does not necessarily require either explicit evaluation of approach- or withdrawal-related action tendencies or linguistic analysis of the words?

In order to address the first question, participants in Study 1 were presented with single written words and asked whether they would approach or withdraw from the concept expressed by each word. We predict faster RTs to PL and NH words than to PH and NL words if valence and arousal contribute to the evaluation of a stimulus' approach and withdrawal tendencies in an interactive manner, as suggested by Robinson and colleagues. If, on the other hand, arousal contributes to the evaluation of a stimulus' approach and withdrawal tendencies linearly for positive and negative stimuli, faster RTs to highly arousing words (PH and NH) than to low-arousal words (PL and NL) will be expected. We also predict that participants will more often decide to approach positively valence words than negative ones and to withdraw from negative words than positive ones. This response pattern should be independent from the arousal dimension of the stimuli and is based on empirical research that showed higher cognitive accessibility of the valence than the arousal dimension (Nicolle and Goel, 2012).

In order to address the second experimental question, participants in Study 2 were asked to respond to each word by either pressing an upper or lower button on the keyboard. Here, reaction tendencies were implicitly associated with spatial information, which has previously been shown to carry implicit embodied meaning: in fact, positive and negative verbal information is conceptually associated with vertical position in space, i.e., with upper vs. lower space, respectively (e.g., *to cheer up, to feel down*; Lakoff and Johnson, 1980). In particular, Meier and Robinson (2004) presented emotionally valenced words either at the top or at the bottom of a computer screen and asked participants to judge their valence (positive or negative) by pressing one of two buttons. They showed faster RTs to congruent stimuli, i.e., to positive than negative words when presented in the upper position, and to negative than positive words when presented at the bottom of the screen. This result demonstrates activation of a conceptual mapping between valence and vertical position, and was replicated in a more implicit task, i.e., responding to target letters in different positions, after having judged the words' valence (see also Rotteveel and Phaf, 2004; Casasanto and Dijkstra, 2010 for associations between valence and vertical position). In line with these findings, we predict that participants will more often respond to positive words by pushing the upper button and to negative words by pushing the lower button.

Most importantly, if implicit approach and withdrawal tendencies are primed automatically during reading and modulated by valence and arousal as suggested by Robinson and colleagues, participants' decisions should be faster for PL and NH words than for PH and NL words. If, on the other hand, stimulus arousal contributes to the priming of approach and withdrawal tendencies linearly for positive and negative stimuli, faster RTs to high-arousal words (PH and NH) than to low-arousal words (PL and NL) will be expected.

## Study 1: Explicit approach and withdrawal task

### Methods

#### Ethics statement

The present studies (1–3) were approved by the Ethics Committee of the Free University of Berlin and are in line with the guidelines of the American Psychological Association. All participants gave written informed consent before taking part in any of the experiments, in accordance with the Declaration of Helsinki.

#### Participants

Nineteen native speakers of German were recruited (16 women, 3 men; age range: 21–67 years, *M* = 33, *SD* = 12). Participants were all right-handed except one and had normal or corrected-to-normal vision; 12 of them were students and seven were workers. They were either paid 5€ or given course credit for their participation.

#### Materials

One hundred and sixty German nouns were selected from the BAWL-R (Võ et al., 2009): 40 positive, high-arousal words (PH), 40 positive, low-arousal words (PL), 40 negative, high-arousal words (NH), and 40 negative, low-arousal words (NL). Word examples and descriptive statistics are reported in Tables 1, 2, respectively. A full list of the stimuli can be found in Appendix A of the Supplementary Material. Words in the four conditions were matched for length in letters, phonemes, and syllables, logarithm of frequency of use, neighborhood (N) size and frequency, and imageability [all *F*s_(3, 156)_ < 1.03, *ns*], according to the values provided by the BAWL-R.

**Table 1:** **Examples of stimuli used for all studies, broken down by condition**.

**Condition**	**Word stimuli**	
	**German (orig.)**	**English (trans.)**
Positive valence, high arousal	SCHATZ	TREASURE
	SPAß	FUN
	BEFREIUNG	LIBERATION
	VORSPIEL	FOREPLAY
Positive valence, low arousal	ERHOLUNG	REGENERATION
	WELPE	PUPPY
	ZUHAUSE	HOME
	BLÜTE	BLOSSOM
Negative valence, high arousal	SCHRECK	FRIGHT
	PISTOLE	GUN
	ERDBEBEN	EARTHQUAKE
	ÄRGER	TROUBLE
Negative valence, high arousal	UNRUHE	AGITATION
	STAU	CUE
	MIETE	RENT
	ÜBELKEIT	NAUSEA

**Table 2:** **Descriptive statistics of affective and psycholinguistic properties of the words included in each condition**.

**Variables**	**Conditions—Mean (SEM)**			
	**PH**	**PL**	**NH**	**NL**
Emotional valence	1.87 (0.07)	1.88 (0.06)	–1.73 (0.05)	–1.74 (0.04)
Arousal	3.47 (0.07)	1.91 (0.05)	4.05 (0.03)	3.21 (0.05)
Imageability	4.26 (0.17)	4.51 (0.21)	4.38 (0.19)	4.04 (0.22)
Frequency of use	24.94 (3.90)	20.60 (3.02)	31.90 (9.55)	26.66 (4.35)
Log frequency	1.12 (0.09)	1.09 (0.08)	1.11 (0.09)	1.03 (0.11)
Letters	6.28 (0.27)	6.38 (0.23)	6.60 (0.21)	6.53 (0.29)
Phonemes	5.65 (0.23)	5.50 (0.21)	5.4 (0.21)	5.68 (0.25)
Syllables	2.05 (0.12)	2.13 (0.11)	1.95 (0.11)	2.18 (0.12)
N-Size	1.70 (0.39)	1.28 (0.29)	1.05 (0.26)	1.23 (0.33)
N-Frequency	294.70 (248.40)	107.78 (76.46)	34.45 (17.78)	365.57 (273.10)

*PH, positive valence, high arousal; PL, positive valence, low arousal; NH, negative valence, high arousal; NL, negative valence, low arousal; SEM, standard error of the mean; N-Size/Frequency, neighborhood-size/frequency*.

**Figure 1:**
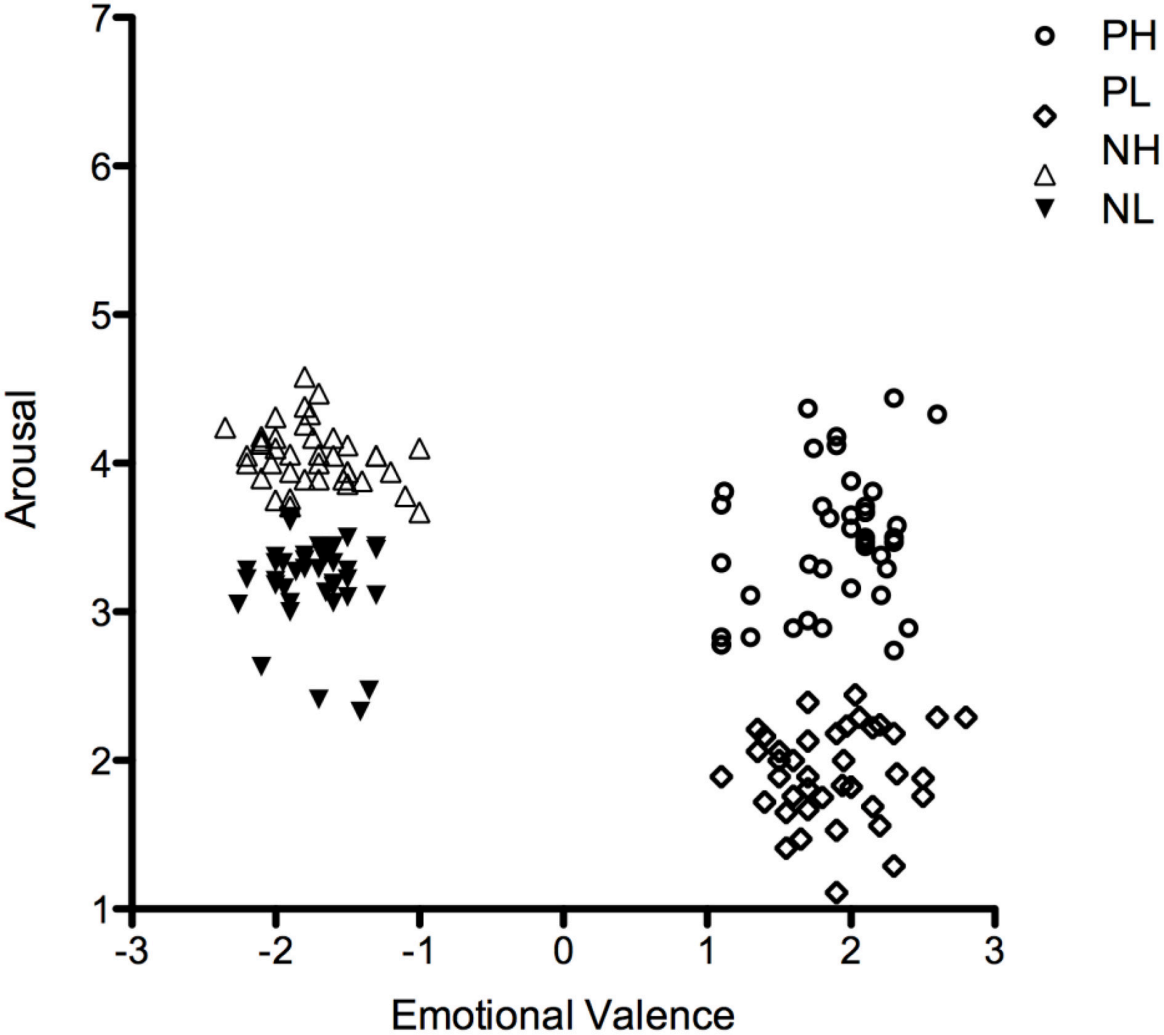
**Distribution of stimulus ratings of emotional valence and arousal (from the BAWL database) for the four experimental conditions: positive high-arousal (PH), positive low-arousal (PL), negative high-arousal (NH), and negative low-arousal (NL)**.

PH words had significantly higher arousal ratings than PL words [*t*_(78)_ = 17.54, *p* < 0.0001], but PH and PL did not differ in valence [*t*_(78)_ = 0.10, *ns*]. Similarly, NH words had significantly higher arousal ratings than NL words [*t*_(78)_ = 14.61, *p* < 0.0001], but did not differ in valence [*t*_(78)_ = 0.18, *ns*]. As can be seen in Figure 1, PH and NH as well as PL and NL words were not exactly matched for arousal because negative words tend to naturally be more arousing than positive words (e.g., Võ et al., 2009; Montefinese et al., 2013; Citron et al., 2014b; Schmidtke et al., 2014). In addition, regarding stimulus arousal, positive words were distributed within a greater range than negative words. This stimulus selection was intended to mimic the natural distribution of affective ratings of words.

#### Procedure

The experiment was programmed with Presentation software (Neurobehavioral Systems, Inc.) and run on a desktop computer. Participants were seated in front of the monitor (monitor screen size 15 inches) at a distance of ~70 cm. The stimuli were presented in the center of the screen in capitalized white letters on a black background (25-point Arial font).

Participants were asked to silently read single words (nouns) that could describe events, sensations, objects, or abstract things (Table 1), and to decide for each word whether they would approach it or withdraw from it. Two response buttons on a German keyboard (F and J, i.e., left and right) corresponded to “approach” vs. “withdrawal” responses. The response buttons were counterbalanced across participants to control for any implicit relations between the valence of the word (positive or negative), the instructions, and the response given (left vs. right): thus, each type of response would be given by half of the participants on one side and by the other half on the opposite side. Key presses had to be made using the index fingers of the right and left hand.

At the start of each trial, a fixation cross appeared in the center of the screen for a variable duration between 400 and 800 ms, followed by a word which remained either until participants gave a response or for a maximum duration of 2000 ms. The screen was then blank for 600 ms; after that, a new trial would start. This short inter-stimulus interval (ITI) was chosen in order to prompt rapid decisions that prevent participants from reflecting too much about the meaning of the stimulus. Stimuli were presented randomly in order to avoid carryover effects from one trial to the other.

A fifteen-trial practice run was followed by an experimental run divided into two sessions with a short break in between. The experimental run contained three filler words at the beginning (which were not used in the analyses) and 160 target words. Word order and condition order (i.e., PH, PL, NH or NL) were pseudo-randomized across participants, i.e., we made sure the same condition would not occur more than three times consecutively in order to avoid carryover effects for a specific emotional category (e.g., Võ et al., 2009; Citron et al., 2014b; Schmidtke et al., 2014). RTs and accuracy (i.e., % of approach vs. total key presses) were recorded for each item. The experiment lasted ~15 min.

#### Data analysis

For each participant, outlying RTs exceeding ±3 *SDs* from the participant's mean RT, as well as trials with no response, were excluded from the analysis, i.e., 1.5% of trials overall. Mean RTs, mean percentage of approach divided by total responses, and *SDs* for each participant and each condition (i.e., PH, PL, NH, and NL), as well as for each stimulus, were calculated. As a standard procedure in psycholinguistic research, we performed all inferential statistical analyses by participant and by item, in order to consider both sources of variability (Clark, 1973). The results of the analyses by item should confirm those obtained in the analyses by participant and allow generalization of the findings on the specific word sample to a broader set of words. However, given the large number of variables that influence word recognition (length, frequency, etc.), item analyses tend to show less significant or weaker effects than the participant analyses. Discrepancies between the two will index less robust effects. As such, confirmation of the findings through careful control for possibly confounding variables will strengthen the reliability of the findings.

For both dependent variables, ANOVAs by participant (indexed by a subscripted 1) and by item (subscripted 2) were conducted, with factors Valence (positive, negative) × Arousal (high, low). Effect sizes were calculated for significant effects and reported as Pearson's *r* coefficients: 0.10 ≤ *r* < 0.30 represents a small effect size, 0.30 ≤ *r* < 0.50 medium and *r*≥0.50 large.

### Results

#### Reaction times

**Figure 2:**
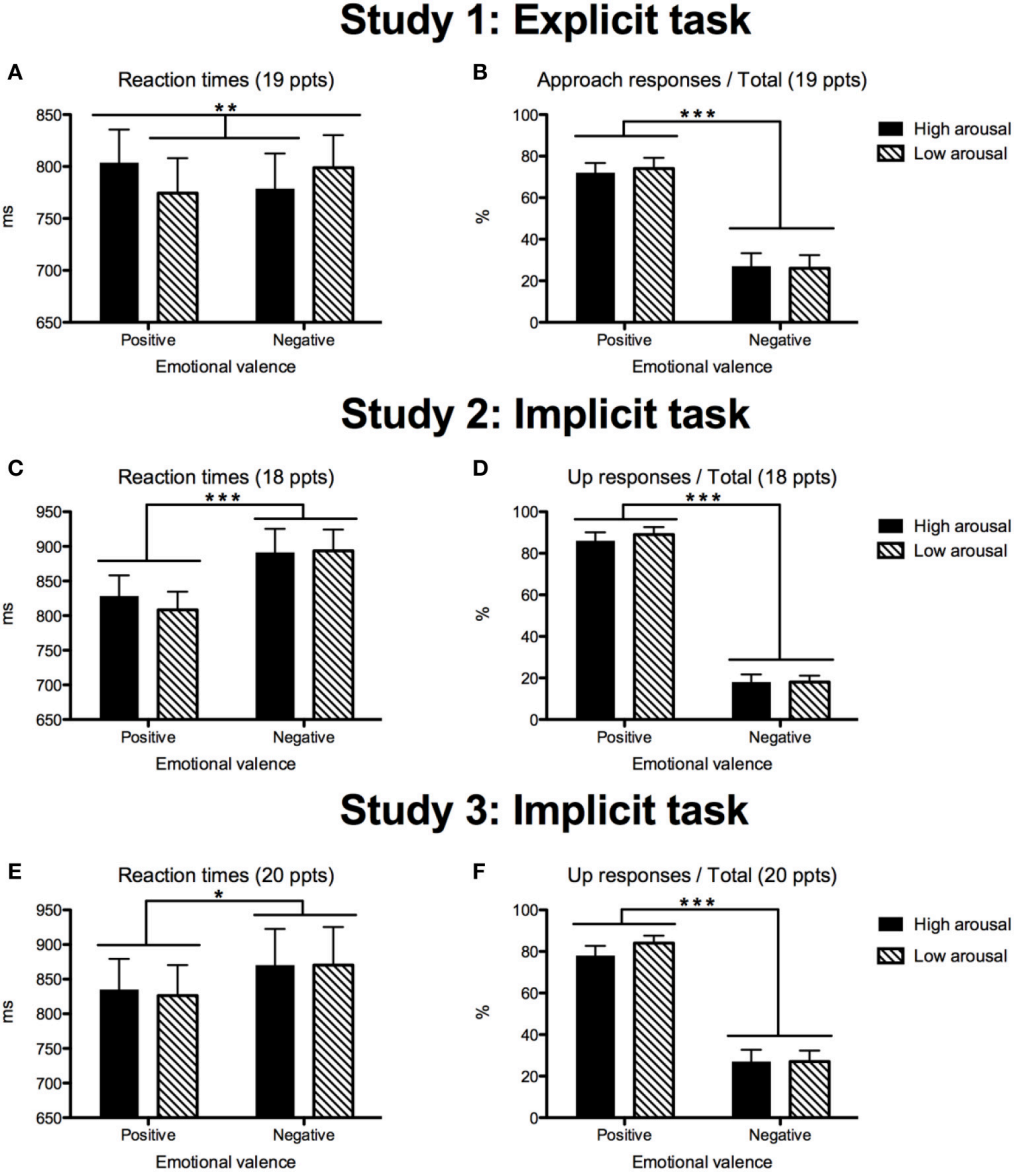
**Descriptive statistics (means + 1 standard error) of reaction times (in ms) and type of response (in percentages) for Study 1 (A,B), Study 2 (C,D), and Study 3 (E,F)**. The statistics are based on the analyses by participant. Significance levels are marked with stars: ^*^p < 0.05; ^**^p < 0.01; ^***^p < 0.001.

A significant interaction between the factors “Valence” and “Arousal” was found [*F*_1(1, 18)_ = 14.93, *p* = 0.001, *r* = 0.67; *F*_2(1, 156)_ = 6.23, *p* = 0.014, *r* = 0.62]. PH and NL words were responded to more slowly than PL and NH words (Figure 2A). No main effects of “Valence” [*F*_1(1, 18)_ = 0.001, *ns*; *F*_2(1, 156)_ = 0.04, *ns*] or “Arousal” [*F*_1(1, 18)_ = 1.25, *ns*; *F*_2(1, 156)_ = 0.31, *ns*] were found.

#### Response type

As predicted, we found a main effect of “Valence” [*F*_1(1, 18)_ = 18.19, *p* = 0.0001, *r* = 0.71; *F*_2(1, 156)_ = 745.90, *p* = 0.0001, *r* = 0.91]. Participants pushed the button corresponding to “approach” more often to positive than negative words and vice versa. No effect of the factor “Arousal” and no interaction between the factors “Valence” and “Arousal” [*F*s_1(1, 18)_ < 1.48, *ns*; *F*s_2(1, 156)_ < 1.21, *ns*] were found (Figure 2B).

## Study 2: Implicit approach and withdrawal task

### Methods

#### Participants

Twenty native speakers of German from the Berlin area were initially recruited. None of them took part in Study 1. The data from two participants had to be excluded from the analyses because they responded “up” to all trials and had extremely fast RTs, possibly suggesting that they did not follow the instructions. The remaining 18 participants (8 women, 10 men; age range: 19–67 years, *M* = 35, *SD* = 12) were all right-handed and had normal or corrected-to-normal vision; eight of them were students, eight were workers, one was unemployed, and one retired. They were either paid 5€ or given course credit for their participation. The excluded participants were right-handed female students, aged 30 and 27 years.

#### Materials and procedure

The linguistic material was exactly the same as in Study 1. The procedure was identical except for the type of task and the response buttons. For the responses, the number keyboard was used: the number 8 was covered with an arrow pointing upwards and the number 2 with an arrow pointing downwards. Participants were asked to keep their index finger on the central button (number 5, covered with paper) and then to spontaneously decide whether to move it upward to press the “up” button or downward to press the “down” button as soon as they saw a word. After each response, they were required to locate their finger on the central button again, in order to avoid facilitation or cost effects due to the position chosen for the last response. Since reading occurs automatically and even against one's own intention (Stroop, 1935), we expected that participants will read the words before responding. Words were presented with the same duration and ITI as in Study 1.

#### Data analysis

The statistical analyses were the same as in Study 1. Detection of outliers led to the exclusion of 2.3% of trials overall.

### Results

#### Reaction times

A main effect of “Valence” was found [*F*_1(1, 17)_ = 18.64, *p* = 0.0001, *r* = 0.72; *F*_2(1, 156)_ = 63.26, *p* < 0.0001, *r* = 0.54]: overall, positive words were responded to faster than negative words. No effect of arousal was found [*F*_1(1, 17)_ = 1.76, *ns*; *F*_2(1, 156)_ = 0.50, *ns*]. In contrast to Study 1, no interaction between the factors “Valence” and “Arousal” was observed [*F*_1(1, 17)_ = 2.49, *p* = 0.13 *r* = 0.36; *F*_2(1, 156)_ = 1.75, *p* = 0.19, *r* = 0.10; Figure 2C].

#### Response type

There was a main effect of valence [*F*_1(1, 17)_ = 113.06, *p* < 0.0001, *r* = 0.93; *F*_2(1, 156)_ = 1782.44, *p* = 0.0001, *r* = 0.96]: participants responded by pushing the “up” button more often to positive than negative words and vice versa. No effect of arousal and no interaction were found [*F*s_1(1, 17)_ < 1.56, *ns*; *F*s_2(1, 156)_ < 1.28, *ns;* Figure 2D].

## Study 3 (follow up on study 2): Implicit task with different instructions

RTs were slower in the implicit task compared to the explicit task (Figures 2A,C), which may reflect larger uncertainty regarding the type of response to be given. However, in contrast to the explicit task, no interactive effects of stimulus valence and arousal were found. This suggests that when participants are unaware of the association between stimulus content and response (“good is up” and “bad is down”), responses are based on stimulus valence, and stimulus arousal does not have any additional effect on response selection. In order to further investigate this, we conducted a third study in which the instructions of the implicit task were slightly modified in order to ensure that participants would fully read the words before responding.

### Methods

#### Participants

Twenty native speakers of German from the Berlin area were recruited (16 women, 4 men; age range: 23–58 years, *M* = 33, *SD* = 12). None of them took part in either Study 1 or 2. Participants were all right-handed and had normal or corrected-to-normal vision; 13 of them were students and seven were workers. They were either paid 5€ or given course credit for their participation.

#### Materials and procedure

The linguistic material used was exactly the same as in Studies 1 and 2 and the procedure identical to Study 2, except for a slight variation in the instructions. In Study 3, participants were asked “to read each word” (while keeping their index finger on the central button, as previously) “and then” to spontaneously decide whether to move their finger upward to press the “up” button or downward to press the “down” button. At the end of the experiment, we also asked participants whether they used any particular strategy for their response decisions.

#### Data analysis

The statistical analyses were the same as in Studies 1 and 2. Detection of outliers led to the exclusion of 0.7% of trials overall.

### Results

#### Reaction times

We confirmed a main effect of “Valence:” as in Study 2, positive words were responded to faster than negative words [*F*_1(1, 19)_ = 4.90, *p* < 0.05, *r* = 0.45; *F*_2(1, 156)_ = 18.09, *p* < 0.0001, *r* = 0.32]. Akin to Study 2, no effect of “Arousal” [*F*_1(1, 19)_ = 0.96, *ns*; *F*_2(1, 156)_ = 0.26, *ns*] and no interaction between the factors “Valence” and “Arousal” were observed [*F*_1(1, 19)_ = 0.58, *ns*; *F*_2(1, 156)_ = 0.003, *ns;* Figure 2E].

#### Response type

The analyses showed a main effect of “Valence:” [*F*_1(1, 19)_ = 50.43, *p* < 0.0001, *r* = 0.85; *F*_2(1, 156)_ = 6.72, *p* < 0.01, *r* = 0.20], no effect of “Arousal” [*F*_1(1, 19)_ = 2.16, *ns*; *F*_2(1, 156)_ = 0.18, *ns*], and no interaction between the factors “Valence “and “Arousal” [*F*_1(1, 19)_ = 3.05, *p* = 0.10; *F*_2(1, 156)_ = 1.04, *ns;* Figure 2F].

At the end of the experiment, upon enquiry by the experimenter most of the participants reported having associated positive words with upper position and negative words with lower position during the task.

## Analysis of potentially confounding factors: Age of acquisition and familiarity

Two further factors need a more thorough consideration as they could have influenced the results of the present experiments. Age of acquisition (AoA) and subjective frequency of encounter with a word (often labeled *familiarity*) have been shown to be positively correlated with a word's emotional valence (Kousta et al., 2011; Citron et al., 2014b). These variables had not been matched during stimulus selection as they were not available in the BAWL-R database. In order to reject these variables as possibly confounding factors, we collected AoA and subjective frequency ratings for our experimental stimuli and re-ran the analyses of our three studies by partialling out their effects. A replication of the current results would reject possible confounding effects of these variables in the present study and further strengthen our findings.

### Methods

AoA was rated by 46 native German speakers (36 women, 10 men; age range: 21–66 years, *M* = 32, *SD* = 11) whereas familiarity was rated by a different group of 48 native speakers (37 women, 11 men; age range: 21–66 years, *M* = 31, *SD* = 11), using seven-point Likert scales^1^.

The main analyses of the three experiments presented were conducted again by partialling out the effects of these two variables. Specifically, in the analysis by participant, raw response types and RTs for each participant were regressed onto item AoA and familiarity ratings separately, and then the resulting standardized residuals were used as the dependent variables; in the analysis by item, the two continuous variables were used as covariates. Since Studies 2 and 3 showed the same results, we merged these two data sets in the current analyses.

### Results

#### Explicit task

In the RTs we replicated a significant interaction between “Valence” and “Arousal,” which was marginally significant in the analysis by item [*F*_1(1, 18)_ = 9.87, *p* < 0.01, *r* = 0.59; *F*_2(1, 154)_ = 2.98, *p* = 0.086, *r* = 0.14; Appendix B, a in Supplementary Material]. No main effects of either “Valence” [*F*_1(1, 18)_ = 0.90, *ns*; *F*_2(1, 154)_ = 2.06, *ns*] or “Arousal” [*F*_1(1, 18)_ = 0.67, *ns*; *F*_2(1, 154)_ = 0.02, *ns*] were found.

In the analysis of the type of response, after partialling out the effects of AoA and familiarity, we replicated a main effect of “Valence” [*F*_1(1, 18)_ = 18.94, *p* = 0.0001, *r* = 0.72; *F*_2(1, 154)_ = 692.72, *p* < 0.0001, *r* = 0.90], no effect of “Arousal” and no interaction [*F*s_1(1, 18)_ < 1.04, *ns*; *F*s_2(1, 154)_ < 0.75, *ns;* Appendix B, b in Supplementary Material].

#### Implicit tasks merged

In the RTs, we replicated a significant main effect of “Valence” [*F*_1(1, 37)_ = 14.52, *p* < 0.001, *r* = 0.53; *F*_2(1, 154)_ = 39.74, *p* < 0.0001, *r* = 0.45], no “Arousal” effect [*F*_1(1, 37)_ = 0.01, *ns*; *F*_2(1, 154)_ = 0.01, *ns*], and no interaction [*F*_1(1, 37)_ = 0.43, *ns*; *F*_2(1, 154)_ = 0.67, *ns;* Appendix B, c in Supplementary Material].

In the analysis of the type of response, we replicated a significant main effect of valence [*F*_1(1, 29)_^2^ = 40.51, *p* < 0.0001, *r* = 0.76; *F*_2(1, 154)_ = 463.22, *p* < 0.0001, *r* = 0.87] but no main effect of arousal [*F*_1(1, 29)_ = 0.25, *ns*; *F*_2(1, 154)_ = 0.97, *ns*]. A significant interaction between valence and arousal was only found in the analysis by participant [*F*_1(1, 29)_ = 6.75, *p* < 0.05, *r* = 0.43; *F*_2(1, 154)_ = 1.87, *ns;* Appendix B, d in Supplementary Material].

## Discussion

The present study investigated reaction times and response type to high- vs. low-arousal positive and negative words in order to test the hypothesis that emotional valence and emotional arousal can affect explicit and implicit approach vs. withdrawal tendencies. In line with the idea proposed by Robinson et al. (2004) that stimulus valence (positive vs. negative) and stimulus arousal (low vs. high) can elicit conflict in processing if the two dimensions do not match (i.e., positive in valence but high in arousal, PH, or negative in valence but low in arousal, NL), we found slower reaction times in response to PH and NL compared to PL and NH words. Interestingly, this response pattern was found only in Study 1, in which participants were explicitly asked whether they would approach or withdraw from the presented words. Thus, this finding extends previous research, which used a range of tasks such as valence or arousal judgment, lexical decision, or the Affective Simon task (Robinson et al., 2004; Purkis et al., 2009; Eder and Rothermund, 2010; Citron et al., 2014c), by showing that both valence and arousal affect participants' explicit decisions on whether to approach or withdraw from the stimulus, as predicted by Robinson et al.'s valence-arousal conflict theory.

Nevertheless, we found that the decision to respond “approach” vs. “withdrawal” through button press was mainly driven by the more cognitively accessible dimension of emotional valence (e.g., Nicolle and Goel, 2012). In fact, participants responded “approach” significantly more often to positive words than to negative ones, and vice versa, irrespective of whether words were high or low in arousal. This is line with the idea that the valence dimension of a stimulus is associated with higher-order cognitive and evaluative processes (such as the ones involved in decision making, as in the present task), while arousal is associated with more automatic physiological reactions (e.g., Herbert et al., 2008; Kissler et al., 2009; for an overview, see Citron, 2012), which are less cognitively accessible (Nicolle and Goel, 2012). Thus, it appears that participants' decisions about whether to approach or withdraw from positive and negative stimuli is strongly influenced by the valence of the stimuli, whereas stimulus arousal appears to influence the speed of this decision in case of conflict (i.e., for PH and NL words). According to Robinson et al. (2004), this conflict occurs because arousal by itself carries emotional and motivational information in such a way that stimuli of high arousal are appraised as negative and elicit a withdrawal orientation whereas stimuli of low arousal are more likely appraised as positive, and elicit approach. Thus, conflict processing should primarily affect evaluation speed as reflected in reaction times, which slow down in response to PH and NL words.

Next, we investigated whether the interactive effects of emotional valence and arousal found during the explicit task will still arise during an implicit task which requires neither explicit evaluation of approach or withdrawal action tendencies (Studies 2 and 3), nor deep linguistic analysis of the words (Study 3). The task employed required participants to respond to visually presented words by pressing either an upper or a lower button (with arrows pointing upward vs. downward). This allowed us to test whether positive and negative words would automatically “push” participants' reactions into a specific direction in space that, according to previous research, would be associated with positive (up) or negative (down) meaning. In both studies (2 and 3) participants responded more often with “up” to positive words and with “down” to negative words, confirming that a mapping of valence onto spatial position is automatically activated. Akin to Study 1, we found that the type of response was not affected by the words' arousal level (low vs. high). Moreover, in contrast to Study 1, reaction times did not differ between PH and PL or NH and NL stimuli, suggesting that spontaneous responses to positive and negative words are primarily driven by “up” and “down” decisions, irrespective of the possibly conflicting information elicited by the arousal dimension of the stimulus. Rather, in Studies 2 and 3 the reaction time data showed a main effect of valence: positive words high and low in arousal were responded to faster than high and low-arousal negative words, suggesting no interference of the arousal dimension with evaluation speed. Thus, in order to activate conflict processing between information conveyed by the valence and the arousal dimension, it seems necessary to employ either an explicit approach vs. withdrawal evaluation task, or a task in which stimulus valence is completely irrelevant and a minimal degree of processing depth is required, such as in the Simon task or the LDT (Eder and Rothermund, 2010; Citron et al., 2014c). In the LDT, real words must be distinguished from pseudowords, i.e., orthographically legal letter strings that could be real words but do not possess any meaning in the target language. In this case, a minimum degree of processing depth is required in order to identify the words. Previous research has shown that, during a word identification task, if words are intermixed with non-recognizable stimuli, no effects of a word's emotional content on either early or late ERP components associated with processing of the emotional content of verbal or pictorial stimuli are elicited, despite behavioral responses being at ceiling (Hinojosa et al., 2010). Thus, words can be identified correctly with no necessary access to their affective connotation.

However, one might doubt whether participants' “up” and “down” responses to positive and negative words actually reflect their implicit motivation to approach or avoid, despite a clear response bias to respond “up” to positive words and “down” to negative words, in line with research showing implicit activation of the conceptual mapping of emotional valence onto vertical position (Lakoff and Johnson, 1980; Meier and Robinson, 2004; Rotteveel and Phaf, 2004; Casasanto and Dijkstra, 2010). The task we employed might carry a confound: whereas up and down-pointing arrows may be associated with high and low spatial position, the finger movement needed to press the upper vs. lower button may require finger extension and contraction and therefore be associated with withdrawal (i.e., pushing a concept away) and approach (i.e., pushing a concept toward oneself), respectively (Solarz, 1960; Cacioppo et al., 1993; Chen and Bargh, 1999). Hence, these two possible associations would lead to opposite predictions. Similarly, there exists research showing that limb extension vs. contraction can be associated with opposite tendencies, i.e., approach vs. withdrawal, respectively, depending on whether the task and instructions require an object-centered or participant-centered perspective (see for instance Lavender and Hommel, 2007; Eder and Rothermund, 2008; Seibt et al., 2008) Whether simple finger movements as required in the present study (instead of whole body or arm movements as required in previous studies) are actually associated with such tendencies regardless of their context is unclear. A task that congruently maps vertical spatial position onto approach could employ a vertically oriented axis with a central button and require participants to push either the upper or lower button by moving their whole hand and then pushing the button; in this way, no difference between finger extension or contraction would be present.

Nevertheless, from the present study we can still confidently conclude that the response decision in our implicit tasks is solely driven by the valence dimension (Nicolle and Goel, 2012) and its implicit mapping onto vertical space. Moreover, we can conclude that this decision is not differentially influenced by the arousal dimension (Meier and Robinson, 2004), therefore extending previous research.

So far, no previous study has investigated whether up and down responses are automatically primed by emotional stimuli and how *both* valence *and* arousal dimensions affect participants' responses to emotional stimuli in such tasks. For example, prior studies have either ignored stimulus-response compatibility effects (e.g., see a detailed discussion in Lynott and Coventry, 2014), and manipulated both valence and space associations (e.g., words presented either above or below on a computer screen) without considering arousal, or only compared positive and negative stimuli rated high in arousal with neutral, low-arousal stimuli.

Interestingly, in the implicit tasks positive words were responded to faster than negative ones. At first glance, this result could be due to the fact that finger movements that require stretching of the fingers are generally faster than finger movements that require a flexion of the fingers. An alternative interpretation of this finding comes from Lakens (2012), who proposed a polarity-based framework. According to this framework, various structural dimensions involved in a task can be considered as dimensions with a +polar and a −polar end, such that stimuli that fall under the “high” +polar end of a scale are processed preferentially and significantly faster than stimuli that fall under the “low” −polar end of this scale. A processing advantage for +polar vs. −polar ends has been demonstrated recently for various stimuli including positive (+polar) vs. negative (−polar) stimuli, spatial dimensions (up vs. down), and moral stimuli (e.g., Clark and Brownell, 1975); furthermore, polar elements of a stimulus or a task can be added together and predictions about processing advantages can be made (Lakens, 2012; Lynott and Coventry, 2014). Notably, even the finger movements required in Studies 2 and 3 could be grouped according to the polarity account into +polar and −polar movements. Thus, responses to positive words are characterized by at least two and up to three +polar ends, i.e., positive valence, “up” position, and finger stretching, whereas responses to negative words have 2 up to 3 −polar ends, i.e., negative valence, “low” position, and finger flexion, therefore causing faster RTs for the former stimuli. Still, this reaction time advantage for +polar over −polar stimuli is not affected by arousal.

The successful replication of all of our results (including Studies 1, 2, and 3) after having partialled out possibly confounding effects of additional variables known to correlate with emotional valence strengthens the effects found in our three experiments, although the interactive effect in the explicit task seems to be somewhat less robust than the main effects. However, the replication of the valence effect only in the implicit tasks seems to strengthen the validity of our findings and shows that these are not spurious effects due to imbalance in AoA or familiarity ratings.

Nevertheless, one possible limitation of the present study might concern the stimulus selection. We aimed to reduce the perceived discrepancy between the arousal level of positive and negative words. In fact, in well-matched experimental selections, negative stimuli with high arousal (matched with positive stimuli) may very likely be perceived as only mildly negative; this is because, in our natural environment, negative stimuli have a clearly higher arousal level than positive ones. By mimicking this natural distribution in our experimental manipulation, on the one hand we have the advantage of reducing or eliminating a perceptual bias (Citron et al., 2014a,c) and of using more ecologically valid stimuli. However, on the other hand we also use a numerically unbalanced 2 × 2 manipulation, e.g., PH stimuli do not differ from NH stimuli only in valence, but also in arousal. However, in case of absence of genuine interactive effects in our data, we would expect a very large arousal effect based on the “numerically unbalanced” stimulus manipulation, i.e., a very large RT difference between the NH and PL words (highest and lowest arousal level, respectively), with an advantage for the former condition. This was not the case in our data.

In addition, the lack of neutral stimuli could be considered a weakness as the emotionality of the material comes out as more obvious. The inter-stimulus interval is relatively short, possibly causing transfer effects of affective variables from one trial to the subsequent one. However, our stimuli were randomized differently across participants, so transfer effects cannot be systematic. Furthermore, Studies 2 and 3 would have benefited from an occasional control task to make sure participants were reading the words. Nevertheless, the systematic mapping between valence and vertical position confirms that participants must have read the words. In fact, the participants we excluded from the analyses responded by pressing the same button for all trials. Finally, the participant samples in Studies 1 and 3 have an unbalanced gender proportion (i.e., more women), unlike Study 2. This might limit the comparison across studies and generalization of the results to a larger, balanced population.

Finally, there exists research showing that individual differences in empathy can affect the strength of emotion-embodiment associations. Specifically, faster reaction times to feeding disgusted faces compared to happy or neutral faces and slower feeding times for happy than disgusted or neutral faces were found; crucially, higher scores on an empathy scale among participants led to stronger effects (Ferri et al., 2010). Therefore, in the present study it would have been interesting to investigate how individual differences in empathy would affect the results found. This is something that may be addressed in future research.

To conclude, the present work furthers empirical research on affective processing and our understanding of the interaction between emotional valence and arousal. While prior research showing interactive effects of emotional valence and arousal during explicit as well as implicit tasks assumed that such effects were due to an integration of implicit approach-withdrawal tendencies (Robinson et al., 2004; Larsen et al., 2008; Hofmann et al., 2009; Purkis et al., 2009; Eder and Rothermund, 2010; Feng et al., 2012; Citron et al., 2014a,c; Recio et al., 2014), the present work explicitly tested this assumption and found evidence for this account only in tasks that require explicit approach-withdrawal decisions for positive and negative words but not in implicit tasks characterized by spontaneous decisions (up vs. down) to positive and negative meaning.

### Conflict of interest statement

The authors declare that the research was conducted in the absence of any commercial or financial relationships that could be construed as a potential conflict of interest.

## Footnotes

^1^The scale for AoA had the following intervals: 0–2, 3–4, 5–6, 7–8, 9–10, 11–12, more than 13 years; whereas the scale for familiarity ranged from 1 (I never read/hear this word) to 7 (I very often read/hear this word). Age ranges for AoA were transformed into point values between 1 and 7 for the analyses. Mean AoA and familiarity ratings and SDs across participants were calculated and two continuous variables obtained.

^2^For some participants, it was not possible to calculate the residuals of the logistic regression because they had more than two missing values.
