# Supplementary material for: Approach and Withdrawal Tendencies during Written Word Processing: Effects of Task, Emotional Valence, and Emotional Arousal
Source: Front Psychol. 2016 Jan 6;6:1935. doi: 10.3389/fpsyg.2015.01935 (PMC4701914; doi:10.3389/fpsyg.2015.01935)
Supplement: Supplementary file 1 [file DataSheet1.docx]

Appendix A. Full list of stimuli used in the experiment and the experimental condition they were assigned to.


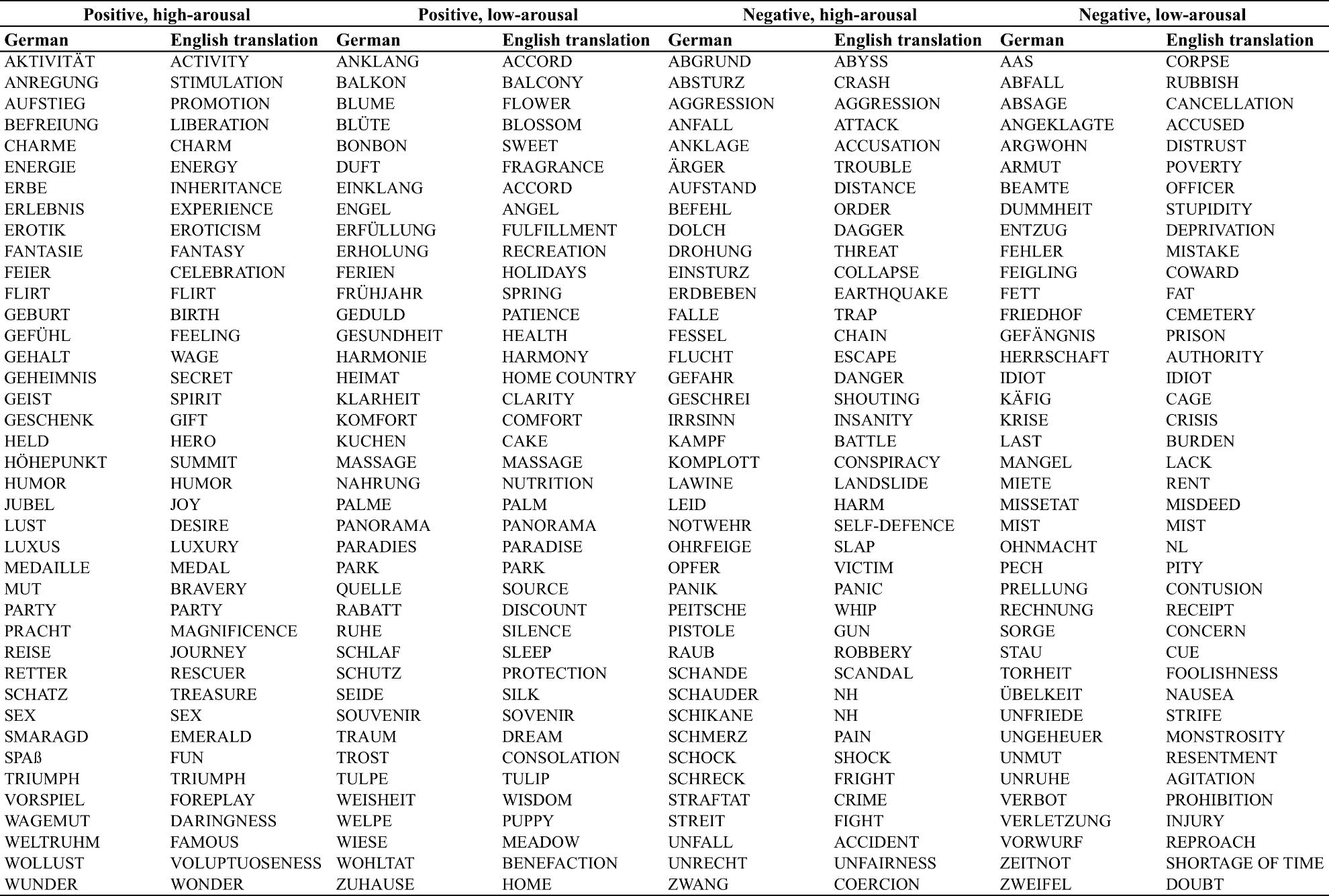


Appendix B.


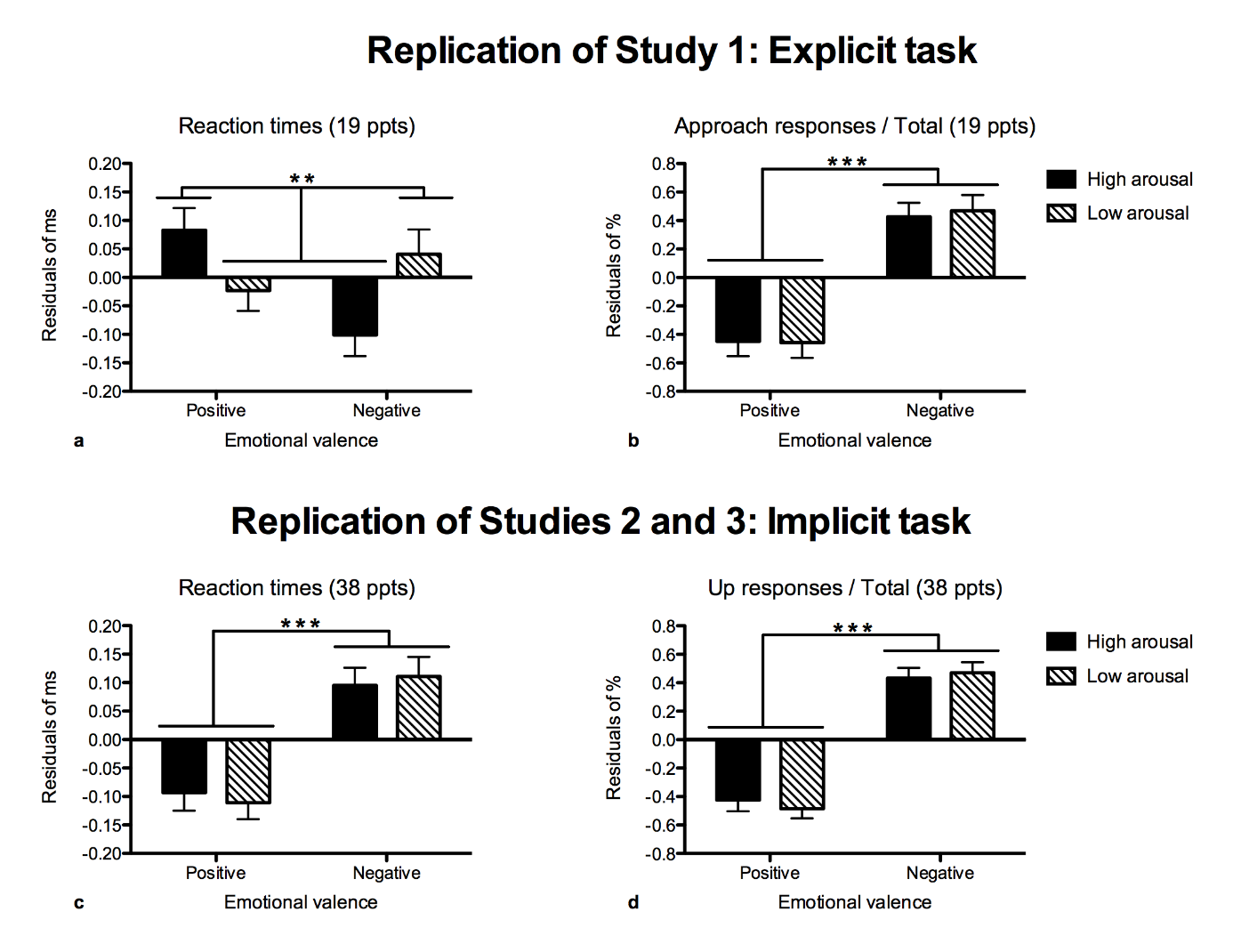


Descriptive statistics (means + 1 standard error) of the resiuals of the reaction times (in ms) and type of response (in percentages) for Study 1 (a, b), and Studies 2 and 3 merged (c, d). In these replications, the variables age of acquisition and familiarity were controlled. The statistics are based on the analyses by participant. Significance levels are marked with stars: * p < 0.05; ** p < 0.01; *** p < 0.001.
